# GP phone calls to improve COVID-19 vaccine uptake among patients at increased risk of severe COVID-19: a randomised trial

**DOI:** 10.3399/BJGPO.2022.0175

**Published:** 2023-04-05

**Authors:** Marit Tuv, Ingeborg Hess Elgersma, Ellen Furuseth, Christine Holst, Arnfinn Helleve, Atle Fretheim

**Affiliations:** 1 Norwegian Institute of Public Health, Oslo, Norway; 2 Centre for Epidemic Interventions Research, Norwegian Institute of Public Health, Oslo, Norway; 3 Division for Infection Control, Norwegian Institute of Public Health, Oslo, Norway; 4 Centre for Evaluation of Public Health Measures, Norwegian Institute of Public Health, Oslo, Norway; 5 Faculty of Health Sciences, Oslo Metropolitan University, Oslo, Norway

**Keywords:** COVID-19, general practitioners, vaccines, primary healthcare, general practice

## Abstract

**Background:**

English media have reported that many unvaccinated individuals took the COVID-19 vaccine after receiving a phone call from their GP.

**Aim:**

To determine whether phone calls from GPs to unvaccinated patients at increased risk of severe COVID-19 improves uptake of the COVID-19 vaccine.

**Design & setting:**

Randomised trial where 202 participants were allocated to receive a phone call from their GP, and 452 participants were allocated to not get the call. Twenty-five GPs at 11 medical centres in Norway took part. The post-trial focus group discussion was with five GPs.

**Method:**

Participants were sourced from the GP electronic medical record system, which communicates with the Norwegian Immunisation Registry and can generate a list of the GPs’ unvaccinated patients at increased risk of severe COVID-19.

**Results:**

The GPs managed to speak over the phone with 154 (76%) patients allocated to receiving a phone call. At follow-up (average 7.5 weeks), 8.9% in the intervention group and 5.3% in the control group had been vaccinated (odds ratio [OR] 1.72; 95% confidence interval [CI] = 0.90 to 3.28). Findings from the focus group discussion suggested the timing of the intervention as a likely key reason for its limited success.

**Conclusion:**

An increase in the proportion of patients who took the COVID-19 vaccine in the intervention group was observed, but the difference was smaller than anticipated, and may be a chance finding. The effect of this type of intervention will likely vary across contexts and may have proved more effective if a larger proportion of the population were unvaccinated.

## How this fits in

According to reports in the English media, a phone call from their GP led many unvaccinated individuals to take the COVID-19 vaccine. Earlier studies have identified personalised phone calls as a measure that may increase influenza vaccine coverage. The findings of the present study indicate that phone calls from GPs had limited effect on vaccination coverage, but the effectiveness was likely influenced by the timing of implementation and the composition of the target group.

## Introduction

Wide uptake of COVID-19 vaccination among groups at increased risk of severe COVID-19 is likely to reduce the incidence of severe disease and death.^1^ Although 94% of Norwegians at moderate or high risk of severe COVID-19 disease had taken at least one dose 1 year after the roll-out of the COVID-19 vaccines, a small but significant proportion of the Norwegian population had not accepted the offer to be vaccinated.^2^ Important factors may include vaccine hesitancy, government distrust, practical barriers, and lack of information.^3^

All Norwegian inhabitants have the right to register with a GP of their choosing. This places GPs in a position as a source of information with potential to influence attitudes towards vaccination, both through leading by example and by conveying clear recommendations about taking the vaccine to their patients.^4–8^

According to media reports of an English pilot scheme, a phone call from their GP led many unvaccinated individuals to take the COVID-19 vaccine.^9^ The authors are not aware of any controlled studies of GPs contacting their patients to increase COVID-19 vaccine coverage, but a systematic review of randomised trials identified several effective measures for improving uptake of influenza vaccines, including postcards, personalised phone calls. and home visits.^10^ To inform decision making around this issue, a randomised trial was conducted among unvaccinated patients at increased risk of severe COVID-19 to see if a phone call from their GP improved vaccination uptake.

## Method

### Trial design

The trial was set up as a two-arm parallel trial, where participants were retrieved from the medical record systems of the GPs who agreed to take part in the study. Patients were individually randomised to one of two parallel groups. The original study protocol (in Norwegian) is available online (DOI:10.5281/zenodo.6381858), as is the data analysis plan (DOI:10.5281/zenodo.6412805). An English translation of the study protocol is included in the Supplementary file.

### Participants and setting

Unvaccinated individuals at increased risk of a severe clinical course of COVID-19 and aged >18 years were eligible. This is in accordance with vaccine eligibility as defined by the Norwegian Institute of Public Health (Folkehelseinstituttet).^11^

The trial started in December 2021, approximately 1 year after the first roll-out of COVID-19 vaccines. At this time, all adults living in Norway had been offered at least two doses of a COVID-19 vaccine, and those defined as at increased risk of severe COVID-19 had been offered three doses. Vaccination was provided conveniently and for free at community vaccination centres, pharmacists, GP offices, and home care services. Although vaccination is voluntary in Norway, there was a high degree of social pressure to be vaccinated, and the vast majority took the first COVID-19 vaccine. Additionally, the authorities launched several campaigns targeted at hard-to-reach groups; for example, certain migrant communities.

A convenience sample of 25 GPs in South-East Norway was recruited to take part in the project. The authors contacted primarily GPs they believed might be willing to take part, loosely based on the authors' own, or others’, understanding.

The GP medical record systems can identify patients at increased risk of clinically severe COVID-19. Moreover, by linking to the Norwegian Immunisation Registry (SYSVAK) the GP systems can differentiate between unvaccinated and vaccinated patients. At each GP office, a GP assistant retrieved a list of unvaccinated patients aged >18 years with an increased risk of severe COVID-19. To limit the burden on participating GPs, it was decided to draw a maximum of 10 patients to the intervention group per GP. Hence, if a GP had fewer than 20 eligible patients, half of them were allocated to the intervention group, and if the number of eligible patients was 20 or more, only 10 were drawn to the intervention group and the remainder were allocated to the control group. The list was stored at the GP office. See further details under *Randomisation* below.

### Intervention

Each participant in the intervention group was contacted by their GP by phone. The control group received no intervention beyond usual care. When the GPs called their patients, they explained the purpose of the phone call (that is, a chance to discuss and ask questions about the vaccine). The GPs emphasised that participation in the project was completely voluntary. The GPs were provided with a 1-page guide for the phone call with patients, and a 2-page document with suggestions on how to address some issues that patients were expected to raise (see Supplementary file).

### Outcome

The only outcome was COVID-19 vaccination; the proportion of participants that had become registered as ‘vaccinated against COVID-19’ in Norwegian Immunisation Registry during the follow-up period was compared between the intervention and control groups.

In practice, outcome measurement was done by repeating the data extraction procedure carried out at the start of the study. At each GP office, a GP assistant retrieved a list of unvaccinated patients aged >18 years with an increased risk of severe COVID-19. By comparing this list with the original list that had been stored in the GP office, the reductions in number of unvaccinated people in the intervention and control groups were determined. The project leader (MT) supervised this process by phone, and reported the figures to the research team’s data analyst (IHE).

### Sample size

An a priori sample size calculation showed that if the proportion of those vaccinated in the intervention group was 20%, and 10% in the control group, the study would need 400 participants with a 1:1 allocation ratio to have 80% power to detect the difference with a statistical significance level of 5%.

### Randomisation

The list of names extracted from each GP’s electronic medical record system was printed out on a spreadsheet with each patient numbered. Up to 10 of the patients on the list were randomly allocated to the intervention group and the remainder to the control group. This was done by using a random number generator (www.gigacalculator.com). By inserting the number of patients on the list and the number of patients to be drawn to the intervention group, the generator yielded a list of random numbers. The patients with these numbers were allocated to the intervention group. The GP assistant highlighted the names of these patients, and then wrote their names on a separate piece of paper. This paper, with the names of the patients in the intervention group, was given to the GP. The spreadsheet with all the extracted names from the medical records system (both intervention and control group patients), was stored in a closed envelope in a locked cupboard at the GP office. The GPs were blinded to the identities of patients in the control group. The project leader (MT) supervised the randomisation process, either by being present at the GP office (for 15 of the GPs) or by phone (for 10 of the GPs). Members of the research team did not see any patient names. All personal data were handled exclusively by employees at the GP office.

### Statistical methods

The analysis adhered to the intention-to-treat principle; that is, the participants were analysed in accordance with the group they were randomised to. Data imputation did not occur. Randomisation was conducted per GP. There is likely statistical dependence between patients who belong to the same GP; this is because these patients share similar environments and because, in Norway, one can choose one’s own GP. Furthermore, GPs may vary in their persuasiveness. To account for this correlation, the data were analysed using a random effects model, where a random intercept per doctor was included (to model correlation between patients) and a random slope of the intervention (to model differences in persuasiveness).

The study used Stata (version 17) and R (version 3.6.3) statistical software. The R-script and dataset are found in the Supplementary file.

### Post-trial focus group discussion

A post-trial digital focus group discussion was convened with five of the GPs who had participated in the study. The aim of the focus group discussion was to gain insights that might explain the trial results by exploring the GPs’ reflections on the intervention and the characteristics of the patient group involved. The focus group discussion was digitally recorded, transcribed, and analysed using an issue-based approach.^12^

### Patient and public involvement

Owing to the nature of this study, including time constraints as a result of the small window of opportunity for carrying out the trial and data privacy constraints, no patients or members of the public were involved in the study design, analysis, interpretation of data, or revision of the manuscript. The project lead (MT) is a practising GP.

### Dissemination to participants and related patient and public communities

All GPs who took part in the study will be sent a summary of trial results and further dissemination will be via a variety of sources, mainly targeting decision makers in Norway, but also towards the public via media outlets.

## Results

### Recruitment and participant flow

**Figure 1.**
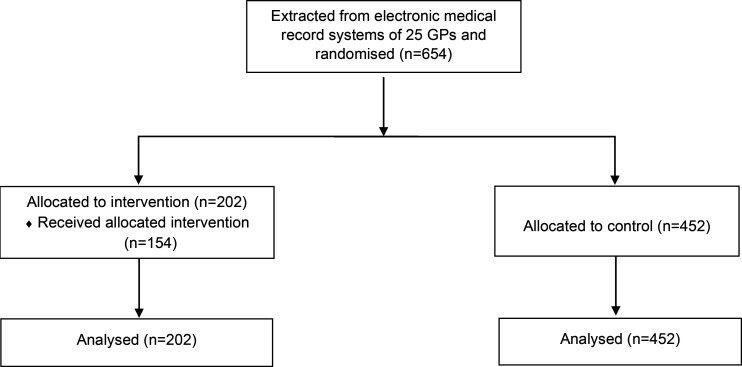
Flow of participants in trial

**Figure 2.**
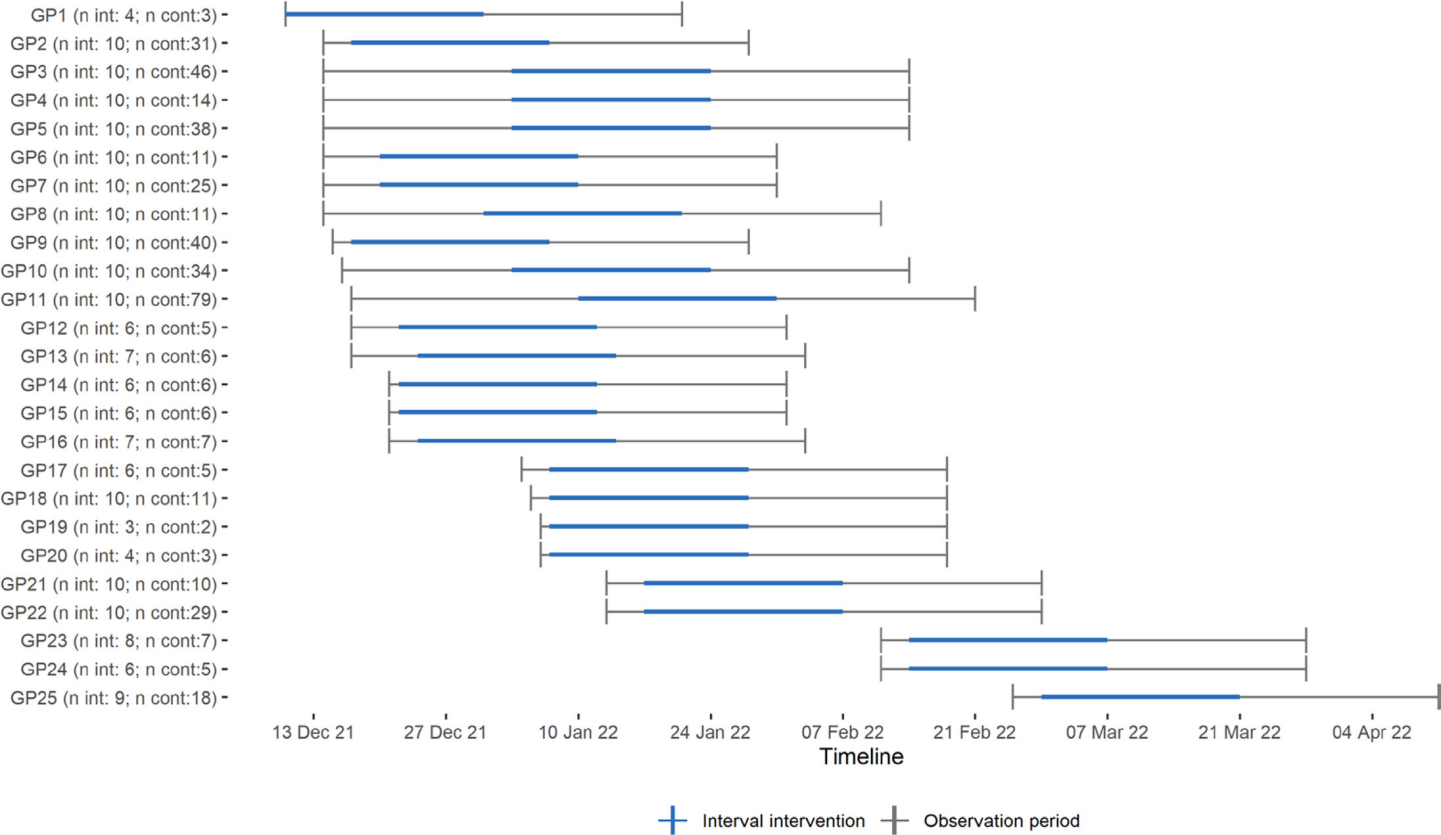
Timeline for each participating GPs and their included patients. Cont = control. Int = intervention.

Figure 1 illustrates the flow of participants through the trial. Twenty-five GPs from a total of 11 GP offices agreed to take part. A total of 654 unvaccinated at-risk patients aged >18 years were retrieved from the electronic medical records system and were included in the trial. The number of included patients per GP varied from 5 to 87 (median 18). In total, 202 patients were allocated to the intervention group and 452 to the control group. The 654 unvaccinated at-risk patients constituted around 2% of all patients on the GPs’ lists. The average follow-up period was 7.5 weeks (range 6.0–9.4) (see Figure 2 for graphic representation). Based on reports from the GPs, it is estimated that they managed to reach 76% (*n* = 154) of the patients they were meant to call.

### Numbers analysed

All 654 randomised participants were included in the analyses.

### Outcomes and estimation

At follow up, 8.9% (*n* = 18/202) in the intervention group and 5.3% (*n* = 24/452) in the control group had been vaccinated (OR 1.72; 95% CI = 0.90 to 3.28) (see Table 1).

**Table 1.** Primary endpoint of the trial. Values are numbers (percentages) of patients

Endpoint	Control (*n* = 452)	Intervention (*n* = 202)	OR (95% CI)
Not vaccinated	428 (94.7)	184 (91.1)	1.00 (reference)
Vaccinated	24 (5.3)	18 (8.9)	1.72 (0.90 to 3.28), *P* = 0.10

CI = confidence intervals. OR = odds ratio.

For most GPs (*n* = 13), none of the patients took a vaccine after receiving a phone call. For seven GPs, one patient took a vaccine after receiving a phone call; for four GPs, two patients took a vaccine; and for one GP, three patients took a vaccine after receiving a phone call.

### Focus group discussion

In the focus group discussion, the GPs expressed the view that the majority of those who were still not vaccinated at the time of the study had either a profound vaccine scepticism or impaired abilities to function in day-to-day life. The GPs all agreed that the intervention was implemented too late in the pandemic, for several reasons. First, the vaccine coverage was already very high at the time of intervention, and only a small group of people at increased risk of severe COVID-19 were not yet vaccinated. Second, the less virulent Omicron variant was already dominating the viral landscape, and it had also recently become known that the vaccine was less efficient in preventing transmission than with previous variants of the SARS-CoV-2 virus. These were the most common reasons that patients gave for not taking the vaccine, according to the GPs. Third, the public discourse around the pandemic and vaccination had changed rapidly before the roll-out of the intervention, and the GPs experienced that many of the patients had already reflected on and made their decision regarding whether to take the vaccine. The GPs experienced some negative responses from patients, typically related to the fact that the patients had been identified as unvaccinated from national health registries. For more details on the focus group discussion findings, see the Supplementary file.

## Discussion

### Summary

This study showed that unvaccinated patients at increased risk of severe COVID-19 were more likely to be vaccinated against COVID-19 if they received a phone call from their GP, but this result is uncertain and may be owing to chance. The vaccination rate in the intervention group was 4.6 percentage points higher than in the control group, which is a smaller effect size than that which the trial was powered to demonstrate.

### Strengths and limitations

It could be argued that the trial was underpowered; however, it is believed an effect size of less than 10 percentage points in vaccination uptake (that is, more than 10 phone calls to achieve one additional vaccinated patient) is unlikely to be worth the effort, considering the cost and limited availability of GP time.

A weakness of the study is that there was very limited data per participant. Therefore, the characteristics of the study population cannot be described (for example, age or country of origin). Also, it cannot be ruled out that some of the study participants had already taken the vaccine without this being reported to the national registry (for example, if the vaccination happened abroad), or that some were erroneously classified being at increased risk of severe COVID-19. A simplistic trial was undertaken for feasibility reasons; for example, avoiding the use of personal data meant that ethical approval and data protection procedures could be expedited. One reason the use of personal data was ethically challenging was that it was practically impossible for written consent to be obtained from all participants. The authors had also planned to ask the GPs to complete a form after each phone call, but decided to sacrifice this data source in order to limit the burden on the GPs and, again, to avoid using personal data.

A major strength of the study is that it was able to evaluate the effectiveness of a policy-relevant intervention within a matter of months, using sound scientific methods, at very low cost. Timeliness and relevance are key factors for research findings to be perceived as valuable for policymakers.^13^ Furthermore, the focus group discussion with participating GPs yielded important insights for the interpretation of the trial results.

The authors cannot exclude that the GPs who took part in the study may have contacted their unvaccinated patients more than an average GP would have done outside a trial setting. This may have occurred for the following two reasons: the participating GPs may have been more positive with regards such interventions than other GPs; and they may have made an extra effort to please the research team.

### Comparison with existing literature

In their Cochrane review on interventions to increase influenza vaccination rates, Thomas and Lorenzetti found that personalised phone calls had *'significant positive effects'* on demand for influenza vaccination.^10^ The present study’s results seem to show little or no effect of phone calls, but there are plausible explanations for this discrepancy, as discussed above. To the authors' knowledge, there have been few, if any, randomised trials of phone calls made by GPs to improve vaccine uptake, and no trials of phone calls to improve COVID-19 vaccination.

### Implications for research and practice

The findings indicate that a phone call from the GP had limited effect on vaccination coverage. The effectiveness of this and similar interventions is likely influenced by the timing of implementation, and the composition of the target group. When the trial was conducted, vaccines had been readily available for 1 year and the vast majority (94%) of the at-risk population had been vaccinated at least once.^2^ Phone calls from GPs may prove more effective in other settings, for example, where a larger proportion of the population has not yet been vaccinated.

## Data availability statement

Our data set is freely available and is included in the Supplementary file.

## Data

The dataset is freely available and is included in the Supplementary file.

## Trial registration number

ClinicalTrials.gov Identifier NCT05207137
